# Impact of Opera on Resilience and Thriving in Serious Mental Illness: Pilot Evaluation of The Center Cannot Hold Part 2 and Resilience Workshop

**DOI:** 10.1007/s10597-024-01248-9

**Published:** 2024-03-22

**Authors:** Kenneth B. Wells, Lily Zhang, Elyn R. Saks, Robert M. Bilder

**Affiliations:** 1 UCLA Jane and Terry Semel Institute Center for Health Services and Society, 760 Westwood Plaza, Suite 17-369B, Los Angeles, CA 90024 USA; 2 David Geffen School of Medicine, Los Angeles, CA 90024 USA; 3 Fielding School of Public Health, Greater Los Angeles Veterans Administration Health System, Los Angeles, CA USA; 4 Center for Health Services and Society, UCLA Jane and Terry Semel Institute for Neuroscience and Human Behavior, Los Angeles, CA USA; 5 Saks Institute for Mental Health Law, Policy and Ethics, Gould School of Law, University of Southern California, Los Angeles, CA USA; 6 Jane & Terry Semel Institute for Neuroscience at UCLA, Los Angeles, CA USA; 7 Stewart & Lynda Resnick Neuropsychiatric Hospital at UCLA, Los Angeles, CA USA

**Keywords:** Arts on schizophrenia recovery, Pilot evaluation, Audience impact

## Abstract

There are few studies of impacts of arts on recovery in schizphrenia, on audience mood and social connection. We developed a pilot evaluation of opera performances in a university setting on Elyn Saks’ journey from psychosis, teaching law and falling in love, coupled with pre-opera workshop on approaches to resilience. Using surveys, primary outcomes were pre and post affect (PANAS-X positive, negative; visual “affect grid” touchscreen for affective valence and arousal) and social connectedness with secondary outcomes of increasing understanding, reducing stigma and willingness to socialize or serve persons with mental illness. Of 107 live and 117 online attendees, 64 completed pre, 24 post, and 22 both surveys. Respondent characteristics were similar for those with pre and pre and post surveys: average age mid 50’s, half female, 10% sexual minority, half White/Caucasian, 13% Hispanic/Latino, 11% Black/African American and 20% Asian; of 22 with pre and post, 9 (41%) were providers. There were significant post–pre increases in positive affect (PANAS-X) and arousal (visual grid) and social connectedness (Cohen’s d = 0.82 to 1.24, each p < .001); and willingness to socialize with someone with schizophrenia (d = 0.68, p = .011). In this pilot evaluation of opera in a university auditorium, despite small sample sizes, there were significant post–pre increases in audience positive affect and social connection, which could reflect selection (those with positive response completing surveys) or may suggest that arts events promote well-being and connection, issues for future larger studies.

**Supplementary Information:**

The online version contains supplementary material available at 10.1007/s10597-024-01248-9.

## Introduction

### Background

The literature on impacts of artworks in therapy and education, based largely on mixed methods, suggests that arts may reduce stigma of mental health conditions (Estroff et al., 2004; Fancourt & Finn, 2019; Heenan, 2006; Lenette et al., 2016; McLean et al., 2011; Ørjasæter et al., 2017; Torrissen, 2015). Further, arts may facilitate understanding or empathy partly by creating audience distance from the subject matter (Ayers et al., 2003; Margrove et al., 2013) while promoting positive atttitudes toward services use (Fancourt & Finn, 2019; Gronholm et al., 2017; Hacking et al., 2006; McLean et al., 2011; Parcesepe & Cabassa, 2013). This is an important issue as stigma may reduce access to care by affecting behavior of those in need, providers, and others (Henderson et al., 2013; Parcesepe & Cabassa, 2013). While effect sizes may be modest or short duration, qualitative studies suggest potential for long-term impact (Michalak et al., 2014). Opera is a complex art form integrating music, poetry and drama, with few studies on impacts (Fancourt & Finn, 2019).

### Context and Framework

Through the Healing and Education through the Arts (HEArts) program (Mango et al., 2018) to develop artworks on mental health, collaborating in evaluation with the UCLA National Endowment for the Arts Research Lab (https://www.NEAResearch.ucla.edu), prior papers describe development and impact of operas on mental health themes, including complicated grief (“The First Lady,” Eleanor Roosevelt after death of Franklin), serious mental illness (“The Center Cannot Hold” on Elyn Saks’ memoire, Saks, 2007) and Veteran post-traumatic stress disorder and homeless (“Veteran Journeys”) (Skrine Jeffers et al., 2022; Bilder et al., 2022, Wells et al., 2021, 2022). Evaluations using pre and post surveys and post-discussion followed a conceptual framework of how artworks engage people in addressing mental health, reduce stigma, increase value of treatment, social support, and empathy, leading to positive affect and social connection (Bilder et al., 2022; Mango et al., 2018). Surveys tracked responses to features of events (opera, pre-event workshop) and characteristics of events, such as a “heroine’s journey” to enhance engagement (Allport, 1954; Patterson & Sextou, 2017; Pinfold et al., 2005; Quinn et al., 2011). Findings included significant post–pre opera increases in audience willingness to engage with persons with grief or schizophrenia, with mediators such as empathy greater for the opera on schizophrenia relative to grief; plus largely positive qualitative comments on audience learning from the opera on schizophrenia (Skrine Jeffers et al., 2022). Similarly, there was a post–pre significant increase in audience willinginess to engage Veterans with post-traumatic stress or unstable housing, including for those reporting personal experience of trauma or unstable housing. Qualitative comments were largely positive, with concerns expressed about viewing trauma and modern compositional style (Bilder et al., 2022). Findings may suggest that opera may be an effective vehicle to promote engagement with mental health issues with increases in positive affect and social connection.

### Goals

Building on this framework and history, the goal was for a pilot evaluation of impact of a new opera presented in a university medical center auditorium on positive recovery from schizophrenia: “The Center Cannot Hold Part II: Recovery” based on Elyn Saks’ memoire (Saks, 2007). The events included a pre-workshop on community-partnered interventions for mental health resilience (Community Partners in Care on collaboration in care for depression, and Together for Wellness/Juntos por Nuestro Bienstar, on digital mental health resources in COVID-19, Wells et al., 2013, 2022). This pilot evaluation included audience pre and post surveys (focus of this paper) and post discussion (future qualitative paper).

### Hypotheses

Consistent with prior studies, we hypothesized that the workshop and opera would increase audience members’ positive affect and sense of social connection as primary outcomes. For secondary/exploratory outcomes, we hypothesized that events would increase views of arts as effective in increasing understanding, reduce stigma of serious mental illness and increase willingness to provide services and make friends/socialize with persons with schizophrenia. We sought to describe audience experience through post survey items. Measures were selected and adapted from prior evaluations on arts on mental health (Bilder et al., 2022; Mango et al., 2018).

## Methods

### Design

This pilot study included pre- and post- surveys available to online registrants and live event attendees through QR codes, with an information sheet online and read at events by an investigator. Survey participation was voluntary and anonymous for persons 18 years or older (exclusion under 18), with data collection approved by UCLA’s Institutional Review Board. The workshop and opera were advertised by UCLA, cast, and community partners and Los Angeles County Department of Mental Health (LACDMH) Take Action LA for Mental Health initiative. Events were free at a medical center auditorium.

### Workshop Format

The 45-min pre-opera workshop on collaboration in resiliency and recovery for diverse populations included remarks by a community facilitator, for 2 events an LACDMH leader, Elyn Saks on her journey to recovery, with remarks by academic and community leads on community-partnered projects on recovery and resilience (Wells et al., 2013, 2022). The workshop concluded with a choral work “I am a Thriver!” by the opera composer based on a poem by community leader Loretta Jones, available on National Academy of Medicine Visualize Health Equity website (https://nam.edu/visualizehealthequity/#/artwork/94).

The Center Cannot Hold Part II: Recovery is a 75-min, one-act opera, with music by a psychiatrist based on memoire by Elyn Saks. The story features her appointment as law professor at USC, experiences of schizophrenia and treatment including conflicts with her provider, different parts of herself represented by 3 characters; and her journey to recovery as a law school professor, falling in love with a law school librarian.

### Pre- and Post-Opera Survey Measures

The pre-opera survey assessed demographics (age, gender identity, race/ethnicity, education, zip code; language preference); Veteran/military status; healthcare, other service, or not provider; and two yes/no questions on ever had or known someone with serious mental illness/schizophrenia or provided services to such persons.

### Primary Outcomes

Measures from the Arts Impact Measurement System (AIMS) included a subset of 4 items from the Positive and Negative Affect Schedule, Expanded Form (PANAS-X) (Watson & Clark, 1994) rated on an ordinal scale (from 1 = very slightly or not at all, to 5 = extremely), for mean of 2 positive items (inspired, proud) and 2 negative items (nervous, distressed), with affect balance ratio as mean of positive divided by mean of negative items. We included a two-dimensional “affect grid”, for which users touched the screen or used a mouse on their device, to indicate on a color-gradient with two axes: (1) affective valence, from negative on left to positive on right (primary); and (2) arousal, from low at bottom to high at top (secondary), converted to affective valence scores and arousal (activation) level scores from 1 to 10, with overall “balance” score. We included revised Social Connectedness Scale (SCS) (Lee & Robbins, 1995) with 6-point responses (1 = strongly disagree to 6 = strongly agree) for 8 aspects of feeling connected rather than disconnected (e.g., society, peers, others) (Bilder et al., 2022).

### Secondary Outcomes

We included: 1) two items (5-point response strongly agree to strongly disagree) assessing if opera/musicals can increase understanding of mental illness and empathy; and reduce stigma of mental illness (mean score); and 2) two items on willingness to be friends or socialize, or support or provide services to someone with serious mental illness/schizophrenia, responses definitely “willing,” to “unwilling” (mean score).

Reliability was assessed with standardized Cronbach’s Alpha (CA) (Cronbach, 1951). The AIMS measures (primary) and 2 understanding/stigma and 2 willingness items (secondary), were repeated in the post-opera survey with post–pre mean difference calculated. All survey items are in Supplemental material (Appendix).

### Exploratory Outcomes (Post-Survey)

The post-survey had items on events attended (workshop, opera live or online; or none); 5-items on arts engagement (5-point strongly agree to strongly disagree response), on being more sympathetic towards persons needing services, more comfortable around those with serious mental illness, less alone with concerns about mental illness, more comfortable talking about it, and reaching out to offer support; 7-items for how well opera and workshop communicated (5 point scale, 5 highest) the importance of social/family support, hope for recovery, a “heroine’s” journey to resilience/recovery, understanding/empathy, helping others, seeking support, and consequences of social stigma, with total score for workshop and for opera; plus overall satisfaction with opera (5-point very to not at all satisfied). The survey had an open-ended response option (for qualitative paper).

### Analysis

We used descriptive univariate and bivariate analyses to compare characteristics of respondents with pre only and pre and post surveys and other comparisons such as by healthcare provider status using Chi-square tests for categorical data and Wilcoxon Mann–Whitney tests [Z] for mean differences. For primary and secondary outcomes, we provide unadjusted tests of post–pre change scores using Wilcoxon 1 sample signed-rank test [W], but discuss multiple comparisons. We used standardized effect sizes (Cohen’s d) for comparisons, calculating mean difference divided by standard deviation of mean difference (Lakens, 2013). We considered as primary, mean change (post–pre) in positive and negative mood, affect grid affect and arousal scores, and social connectedness (5 measures); for secondary, mean score for impact of arts to promote understanding and address stigma of mental health; and mean score for willingness to provide services and make friends/socialize with someone with schizophrenia (2 measures). For exploratory post-only items, we describe impact on 5 “humanistic” arts engagement items and mean, and tested difference in total score for impact ratings of workshop and opera, as a pilot evaluation to inform future research.

## Results

### Audience Characteristics

Of 107 live and 117 online attendees, 64 completed pre, 24 post, and 22 pre and post surveys. As shown in Table 1, there were no significant differences in characteristics measured in pre-survey (demographics, provider status, experiences with serious mental illness/schizophrenia) between those completing only pre-survey (N = 42) and both pre and post (N = 22) (each p > 0.05); and there were also no significant differences on other pre measures including arts engagement, mood and social connection (each p > 0.05). For those with any pre-survey, mean age was mid 50’s, 55.56% female, 41.27% male, and 3.17% identified as transgender/non-binary and 11.74% as sexual minority. Of 61 reporting race/ethnicity, 55.74% identified as White, 13.11% any Hispanic, Latino or Spanish origin, 11.48% as primarily Black/African American or African, and 19.67% as primarily Asian descent. For 92.19%, English only was their preferred language. The majority (73.44%) had graduate school education, and 41.27% were a health care provider. About half reported knowing someone with schizophrenia or serious mental illness (59.38%) or supporting or providing services (44.44%). For primary measures, using pre-survey data, standardized Chronbach’s Alpha (CA) results were: 1) PANAS-X positive mood, CA = 0.86; 2) PANAS-X negative mood, CA = 0.72; 3) Social connectedness, CA = 0.94. For secondary; 1) arts increase understanding/stigma, CA = 0.84; 2) willingness to make friends/provide services, CA = 0.81. For post-survey only comparison of mean for 7 items for workshop and opera impact, standardized CA for workshop = 0.91 and for opera = 0.84; and for mean of 5 arts engagement items, standardized CA = 0.93.

**Table 1:** Characteristics of Survey sample pre-event by status of pre-post completion

Variables	N	Overall N = 64	Pre ONLY N = 42	Both Pre and Post N = 22	p value^†^
Demographics					
Age, in years, Mean (SD)	64	54.52 ± 15.92	56.55 ± 16.31	50.64 ± 14.74	0.210
Gender Identity	63				0.320
Female		35 (55.56)	20 (48.78)	15 (68.18)	
Male		26 (41.27)	19 (46.34)	7 (31.82)	
Other (transgender, non-binary)		2 (3.17)	2 (4.88)	0 (0.00)	
Race/Ethnicity (Hierarchy: H/B/Oth/W)	61				0.636
Any Hispanic/Latino/Spanish heritage		8 (13.11)	5 (12.5)	3 (14.29)	
Black/African American/African (primary)		7 (11.48)	5 (12.5)	2 (9.52)	
Asian (primary)		12 (19.67)	6 (15.00)	6 (28.57)	
White/Caucasian or Middle Eastern descent		34 (55.74)	24 (60.00)	10 (47.62)	
Education	64				0.194
Less than High School or high school graduate	64	–	–	–	
Vocational or Some College		3 (4.69)	3 (7.14)	0 (0.00)	
College Graduate		14 (21.88)	11 (26.19)	3 (13.64)	
Graduate School (J.D., Master's, PhD, MD)		47 (73.44)	28 (66.67)	19 (86.36)	
Sexual minority	62				0.300
No		51 (82.26)	31 (77.5)	20 (90.91)	
Yes		11 (17.74)	9 (22.5)	2 (9.09)	
Preferred language	64				0.329
English only		59 (92.19)	40 (95.24)	19 (86.36)	
Other language		5 (7.81)	2 (4.76)	3 (13.64)	
Health care provider	63	26 (41.27)	17 (41.46)	9 (40.91)	0.966
Experience of schizophrenia					
Someone you know ever had schizophrenia or serious mental illness (Yes/No)	64	38 (59.38)	26 (61.9)	12 (54.55)	0.569
Supported or provided services for someone with schizophrenia or serious mental illness (Yes/No)	63	28 (44.44)	20 (47.62)	8 (38.1)	0.473

Data are presented as mean ± standard deviation (SD) for continuous variables, % for categorical variables, variation in N across variables results from missing data

^†^ Wilcoxon-Mann–Whitney test for continuous variables and Chi-square test or Fisher’s exact test for categorical variables

### Post–Pre Difference

For those having both post and pre surveys, there were significant post–pre differences in PANAS-X mean positive score (and confirmatory affect balance ratio), mean social connectedness scale and positive affect mood grid score (Cohen’s d ranged from 0.96–1.24, each p < 0.001, significant at p < 0.05 adjusting for 5 primary outcomes). Difference in mean arousal score (d = 0.56, p = 0.018) (Table 2) would not be significant adjusting for 5 outcomes. For secondary outcomes, mean change in increasing understanding or reducing social stigma was not significant (0.25 ± 0.69, p > 0.05); mean for making friends or socializing or providing services for someone with schizophrenia was significant (0.34 ± 0.52, p = 0.016), and adjusting for 2 secondary outcomes.

**Table 2:** Post–Pre Survey responses

Variables	N	Pre (Mean ± SD)	Post (Mean ± SD)	Difference (Mean ± SD)	^†^test score [W]	p†	ES^‡^
In your opinion							
Watching an opera or musical can increase understanding of emotional stress	22	4.18 ± 0.80	4.45 ± 0.74	0.27 ± 0.88	16.5	0.243	0.31
The arts can reduce social stigma of mental illness	22	4.50 ± 0.67	4.73 ± 0.46	0.23 ± 0.61	12.5	0.18	0.37
Mean understanding/stigma score	22	4.34 ± 0.68	4.59 ± 0.53	0.25 ± 0.69	25	0.111	0.36
How willing would you be to:							
Make friends or socialize with someone suffering from schizophrenia	22	3.18 ± 0.73	3.64 ± 0.49	0.45 ± 0.67	27.5	0.011	0.68
Support or provide services to someone with schizophrenia	22	3.32 ± 0.99	3.55 ± 0.67	0.23 ± 0.69	10	0.234	0.33
Mean socialize/services score	22	3.25 ± 0.78	3.59 ± 0.55	0.34 ± 0.52	30	0.016	0.65
Positive and negative affect scale							
Mean positive score	22	2.50 ± 0.83	3.73 ± 0.91	1.23 ± 1.12	95.5	< .001	1.10
Mean negative score	22	1.43 ± 0.54	1.27 ± 0.37	-0.16 ± 0.52	19	0.222	0.31
Affect balance ratio	22	1.91 ± 0.77	3.18 ± 1.13	1.27 ± 1.32	87.5	< .001	0.96
Visual affect grid scales							
Mean affective score	21	6.52 ± 1.33	7.76 ± 1.34	1.24 ± 1.00	68	< .001	1.24
Mean arousal score	21	5.05 ± 2.11	6.38 ± 1.94	1.33 ± 2.37	57	0.018	0.56
Social Connectedness Scale total score	22	34.41 ± 7.71	39.23 ± 6.82	4.82 ± 5.88	90	< .001	0.82

^†^Wilcoxon Signed-Rank test

^‡^standardized effect size

### Post only Survey

For exploratory post-only outcomes for 24 with any post data (22 pre and post; 2 post only), 11 reported attending both workshop and opera, 4 one event, and 9 not responding, with no significant differences in post-event engagement measures for those attending both versus one or unknown (each p > 0.10). As shown in Table 3, for comparison of healthcare providers with others, the mean arts engagement score was high across groups (each 4.40) and not significantly different (Z(1) = 0.68, p = 0.499). The total impact score for the workshop was significantly higher for healthcare providers than others (Z(1) = 2.32 p = 0.020) with significant item-level differences for portraying a “heroine’s journey to resilience/recovery” (Z(1) = 2.24, p = 0.025), increasing commitment to help others (Z(1) = 2.19, p = 0.028) and portraying personal consequences of social stigma (Z(1) = 2.89, p = 0.004). In contrast, the total impact score for the opera did not differ significantly for providers and others (Z(1) = 1.72, p = 0.085). For persons without experience with serious mental illness personally or knowing others versus those with such experience, the total impact score for the workshop had a borderline significant trend towards more impact for those with experience (Z(1) = -1.91, p = 0.057), but no significant difference for opera total impact score (Z(1) = -1.28, p = 0.199). Overall satisfaction was high (1.36 ± 0.73) and not significantly different by provider status (Z(1) = -0.21, p = 0.83) or personal experience with serious mental illness (Z(1) = 0.08, p = 0.93).

**Table 3:** Post event only Items and response for HealthCare Providers versus Others

	N	Overall mean ± SD N = 22	Provider mean ± SD N = 9	Other participant mean ± SD N = 13	Test score† Z[1]	p value^†^
The event (workshop and opera) has moved you in (1–5, 5 strongly agree)						
Being more sympathetic	22	4.45 ± 0.67	4.67 ± 0.71	4.31 ± 0.63	1.46	0.143
Feeling more comfortable around persons with serious mental illness	22	4.36 ± 0.66	4.44 ± 0.88	4.31 ± 0.48	0.85	0.394
Feeling less alone with concerns about mental illness	22	4.32 ± 0.84	4.11 ± 1.17	4.46 ± 0.52	-0.33	0.741
Feeling more comfortable talking about mental illness	22	4.32 ± 0.78	4.22 ± 0.97	4.38 ± 0.65	-0.18	0.855
Reaching out more to offer support	22	4.55 ± 0.67	4.56 ± 0.73	4.54 ± 0.66	0.12	0.906
Mean arts engagement measure	22	4.40 ± 0.53	4.40 ± 0.77	4.40 ± 0.32	0.68	0.499
How well the workshop and opera convey each concept (1–5, 5 highest)						
Workshop on Resilience						
Importance of social/family support	21	4.33 ± 0.97	4.56 ± 1.01	4.17 ± 0.94	1.1	0.269
Importance of hope for recovery	21	4.38 ± 0.92	4.78 ± 0.67	4.08 ± 1.00	1.72	0.085
A heroine’s journey to resilience/recovery	21	4.24 ± 1.18	4.89 ± 0.33	3.75 ± 1.36	2.24	0.025
Increase understanding/empathy	21	4.29 ± 0.78	4.56 ± 0.53	4.08 ± 0.90	1.16	0.248
Increase commitment to help others	21	4.33 ± 0.86	4.78 ± 0.67	4.00 ± 0.85	2.19	0.028
Importance of seeking help or support	21	4.48 ± 0.81	4.78 ± 0.67	4.25 ± 0.87	1.62	0.105
Personal consequences of social stigma	21	4.24 ± 0.89	4.89 ± 0.33	3.75 ± 0.87	2.89	0.004
Overall score for workshop on Resilience	21	30.29 ± 5.30	33.22 ± 3.35	28.08 ± 5.53	2.32	0.02
The Center Cannot Hold Part 2: Recovery						
Importance of social/family support	22	4.68 ± 0.72	4.67 ± 1.00	4.69 ± 0.48	0.82	0.411
Importance of hope for recovery	22	4.77 ± 0.43	4.78 ± 0.44	4.77 ± 0.44	0.0	1.00
A heroines journey to resilience/recovery	22	4.68 ± 0.57	4.78 ± 0.44	4.62 ± 0.65	0.47	0.637
Increase understanding/empathy	22	4.73 ± 0.55	4.67 ± 0.71	4.77 ± 0.44	-0.05	0.964
Increase commitment to help others	22	4.41 ± 0.91	4.67 ± 1.00	4.23 ± 0.83	1.64	0.102
Importance of seeking help or support	22	4.68 ± 0.78	4.89 ± 0.33	4.54 ± 0.97	0.74	0.456
Personal consequences of social stigma	22	4.23 ± 1.02	4.56 ± 1.01	4.00 ± 1.00	1.54	.122
Overall score for opera	22	32.18 ± 3.54	33.00 ± 4.24	31.62 ± 3.01	1.72	0.085
Overall, how satisfied were you with the opera performance	22	1.36 ± 0.73	1.44 ± 1.01	1.31 ± 0.48	-0.21	0.83

^†^Wilcoxon-Mann–Whitney test for Likert scale variables

## Discussion

This pilot study evaluated the impact of an arts event (opera) on recovery from schizophrenia, based on lived experiences of a law professor starting teaching and coping with her illness yet falling in love. The opera was preceded by a workshop on community resilience. The events were held in a university medical center auditoirum, with an audience that included many medically trained members and some with lived experience, which could suggest that audience members were familiar with mental health issues. A small number of individuals from a larger audience completed both pre- and post-event surveys. Survey participants may have been more engaged in the event. These issues raise important limitations on findings in terms of generalizability both to broader audiences and settings and interpretation of findings. Despite these limitations there may be implications for future studies examining the impact of arts events related to recovery from severe mental illness. Participant characteristics were similar in demographics, arts engagement, mood and social connection for those with only pre and both pre and post data, suggesting bias due to survey participation might be more related to completing any survey than both surveys. Further, despite a small sample, following the conceptual framework from prior arts evaluations, there were statistically significant increases from pre to post in two measures of positive affect/mood, and feelings of social connection. Serious mental illness is noted as an important health condition (Kessler et al., 2005; Wu et al., 2006), and it is possible that presenting true stories of recovery through art (with the individual/author present) may increase positive affect and sense of belonging. Similarly, for secondary outcome measures, there was an increased willingness to make friends or provide services to people with SMI, significant adjusting for two secondary outcomes, consistent with findings on social connection. Given the location in a healthcare setting and having providers present, we compared providers with others for the opera and pre-opera workshop. While overall engagement in the opera was similar, we found that providers were more strongly engaged than others by the workshop, with a borderline significant similar trend for persons with lived experience personally or knowing others with schizophrenia. This may be consistent with such events being more engaging for audiences familiar with similar education formats or having lived experience with the subject matter. Participatory artworks have been shown to impact stigma, such as “Playback Theater” (Yotis et al., 2017), “Forum Theater” (Wilson, 2013) and knowledge translation in bipolar disorder (Michalak et al., 2014). However, we did not observe a significant effect on the single stigma reduction item in post–pre comparison, which could be due to small sample size or audience prior knowledge of serious mental illness, with moderately high pre-survey response on understanding/view of arts affecting stigma. This effect is important to explore with larger samples with more diverse education and life experiences, as well as broader stigma impact measures. Even in this context, results may suggest increased mood and social connection after art events.

### Limitations

The events were premiers of a one-act opera with pre-workshop, presented in an academic medical center auditorium, with lead character/co-librettist and composer participating in workshops, which could have stimulated positive responses. Sample sizes for surveys were small with high representation of providers or those with lived experience. There were no comparison conditions. However, primary outcomes would be significant adjusting for multiple comparisons, and post measures suggested similar engagement across audiences for the opera, yet more engagement in the workshop for providers. More general audiences could respond differently.

## Conclusion

For this pilot evaluation, with a small sample size in the evaluation from audiences in a medical center auditorium, primary findings on post–pre outcomes are consistent with potential for opera events on true lived experience of serious mental illness to increase positive affect and social connection, with an exploratory finding that educational workshops may be more engaging for providers and persons with lived experience. While characteristics were similar for persons with only pre and pre and post surveys, findings could reflect selection effects with those with more experience and a tendency for positive reaction to such events to complete both surveys. However, findings could reflect actual impact, and may inform future studies of more general audiences, larger survey samples, and as feasible, comparison conditions such as participation in other types of events.

### Supplementary Information

Below is the link to the electronic supplementary material.

Supplementary file1 (DOCX 720 KB)

## References

[CR1] Allport, G. (1954) The Nature of Prejudice Perseus Books. *Cambridge, MA*.

[CR2] Ayers JW, Althouse BM, Leas E (2003). Internet searches for suicide following the release of '13 Reasons Why'. Health.

[CR3] Bilder RM, Mango J, Tang L (2022). Impact of *Veteran Journeys* opera on audience member attitudes related to veterans with post-traumatic stress or homelessness. Psychology of Aesthetics, Creativity, and the Arts.

[CR4] Cronbach LJ (1951). Coefficient alpha and the internal structure of tests. Psychometrika.

[CR5] Estroff SE, Penn DL, Toporek JR (2004). From stigma to discrimination: An analysis of community efforts to reduce the negative consequences of having a psychiatric disorder and label. Schizophrenia Bulletin.

[CR6] Fancourt D, Finn S (2019). What is the evidence on the role of the arts in improving health and well-being? A scoping review.

[CR7] Gronholm PC, Henderson C, Deb T (2017). Interventions to reduce discrimination and stigma: the state of the art. Social Psychiatry and Psychiatric Epidemiology.

[CR8] Hacking S, Secker J, Kent L (2006). Mental health and arts participation: the state of the art in England. The Journal of the Royal Society for the Promotion of Health.

[CR9] Heenan D (2006). Art as therapy: An effective way of promoting positive mental health?. Disability & Society.

[CR10] Henderson C, Evans-Lacko S, Thornicroft G (2013). Mental illness stigma, help seeking, and public health programs. American Journal of Public Health.

[CR11] Kessler RC, Birnbaum H, Demler O (2005). The prevalence and correlates of nonaffective psychosis in the National Comorbidity Survey Replication (NCS-R). Biological Psychiatry.

[CR12] Lakens D (2013). Calculating and reporting effect sizes to facilitate cumulative science: a practical primer for t-tests and ANOVAs. Frontiers in Psychology..

[CR13] Lee RM, Robbins SB (1995). Measuring belongingness: The Social Connectedness and the Social Assurance scales. Journal of Counseling Psychology.

[CR14] Lenette C, Weston D, Wise P (2016). Where words fail, music speaks: The impact of participatory music on the mental health and wellbeing of asylum seekers. Arts & Health.

[CR15] Mango JD, Saks E, Skrine Jeffers K (2018). Addressing mental health stigma through the arts: an academic-community partnered program. The Behavior Therapist.

[CR16] Margrove KL, Pope J, Mark G (2013). An exploration of artists' perspectives of participatory arts and health projects for people with mental health needs. Public Health.

[CR17] McLean J, Woodhouse A, Goldie I (2011). An evidence review of the impact of participatory arts on older people.

[CR18] Michalak EE, Livingston JD, Maxwell V (2014). Using theatre to address mental illness stigma: A knowledge translation study in bipolar disorder. International Journal of Bipolar Disorders.

[CR19] Ørjasæter KB, Stickley T, Hedlund M (2017). Transforming identity through participation in music and theatre: exploring narratives of people with mental health problems. International Journal of Qualitative Studies on Health and Well-Being.

[CR20] Parcesepe AM, Cabassa LJ (2013). Public stigma of mental illness in the United States: A systematic literature review. Administration and Policy in Mental Health and Mental Health Services Research.

[CR21] Patterson P, Sextou P (2017). ‘Trapped in the labyrinth’: Exploring mental illness through devised theatrical performance. Medical Humanities.

[CR22] Pinfold V, Thornicroft G, Huxley P (2005). Active ingredients in anti-stigma programmes in mental health. International Review of Psychiatry.

[CR23] Quinn N, Shulman A, Knifton L (2011). The impact of a national mental health arts and film festival on stigma and recovery. Acta Psychiatrica Scandinavica.

[CR24] Saks ER (2007). The center cannot hold: My journey through madness.

[CR25] Skrine Jeffers K, Mango JD, Tang L (2022). Impact of opera on mental health stigma: pilot of provider/community workshop. Community Mental Health Journal.

[CR26] Torrissen W (2015). ‘Better than Medicine’: Theatre and health in the contemporary norwegian context. Journal of Applied Arts & Health.

[CR27] Watson, D., & Clark, L. A. (1994). The PANAS-X: Manual for the positive and negative affect schedule-expanded form. 10.17077/48vt-m4t2

[CR28] Wells KB (2022). Veteran journeys: creating an opera based on research, clinical, and personal experiences. Psychiatric Services.

[CR29] Wells KB, Jones L, Chung B (2013). Community-partnered cluster—randomized comparative effectiveness trial of community engagement and planning or resources for services to address depression disparities. Journal of General Internal Medicine.

[CR30] Wells KB, Skrine Jeffers K, Mango J (2023). Integration of arts and health sciences in developing an opera on veteran resilience and recovery. Health Promotion Practice.

[CR31] Wells KB, Thames AD, Young, (2022). Together for wellness/juntos collaborators and writing group. engagement, use, and impact of digital mental health resources for diverse populations in COVID-19: community-partnered evaluation. JMIR Form Res.

[CR32] Wilson, J.J. (2013) Let’s all play ‘Stigma’! Learning together using forum theatre in collaboration with mental health service users and nurse lecturers. *Working Papers in Health Sciences 1*(4), 1–3.

[CR33] Wu EQ, Shi L, Birnbaum H (2006). Annual prevalence of diagnosed schizophrenia in the USA: A claims data analysis approach. Psychological Medicine.

[CR34] Yotis L, Theocharopoulos C, Fragiadaki C (2017). Using playback theatre to address the stigma of mental disorders. The Arts in Psychotherapy.

