# Supplementary material for: Impact of Opera on Resilience and Thriving in Serious Mental Illness: Pilot Evaluation of The Center Cannot Hold Part 2 and Resilience Workshop
Source: Community Ment Health J. 2024 Mar 22;60(5):964–71. doi: 10.1007/s10597-024-01248-9 (PMC11169011; doi:10.1007/s10597-024-01248-9)
Supplement: Supplementary file 1 — Supplementary file1 (DOCX 720 KB) [file 10597_2024_1248_MOESM1_ESM.docx]

**The Center Cannot Hold Part 2: Recovery and pre-Event Workshop on Resilience: Pre-Survey**

Thank you for participating in our survey. We ask for background information and views on use of arts in addressing symptoms and treatment for severe mental illness. There is no right or wrong answer and participation is voluntary.

We appreciate your time and support!

**What is your age in years? _________**

**(If under 18: Thank you, you are not eligible for this survey but still may attend the opera! Thank you).**

**If 18 or older: You are eligible. Please continue)**

**Tell Us About Yourself: For the survey below, you have the option of skipping an item if you do not want to answer and continuing with the survey. Thank you!**

1. **What is your zip code?**
   1. [                 ]
2. **Please select your highest level of completed education (select 1):**
   1. Some High School or less than High School
   2. High School Graduate or equivalent (i.e., GED)
   3. Vocational/Certificate Program
   4. Some College
   5. College Graduate
   6. Graduate School (J.D., Master's, PhD, MD)
3. **How would you describe yourself? (check all that apply)**
   1. [ ] American Indian/ Native American/ Alaskan Native
   2. [ ] Black/African American/African
   3. [ ] East Asian (Japanese, Korean, Chinese)
   4. [ ] South East Asian (Vietnamese, Filipino/a, Laotian, Thai, Indonesian, Cambodian)
   5. [ ] South Asian (Indian, Nepali, Bangladeshi, Afghani, Bhutan, Pakistani, Sri Lankan, other)
   6. [ ] Hispanic, Latino or Spanish Origin (Cuban, Mexican, Puerto Rican, South or Central American or other Spanish culture or origin, regardless of race)
   7. [ ] Middle Eastern
   8. [ ] Pacific Islander (Hawaiian, Guamanian, Samoan, Tongan)
   9. [ ] White/ Caucasian/ European
   10. [ ] Unknown
   11. [ ] Prefer not to state
4. **Which BEST describes your gender? (check all that apply)**
   1. Female
   2. Male
   3. Genderqueer
   4. Questioning
   5. Trans Man
   6. Trans Woman
   7. Other gender not listed : _____________
   8. Prefer not to state
   9. Don’t know
5. **Do you think of yourself as straight or heterosexual, gay, lesbian, or homosexual, or bisexual?**
   1. Straight
   2. Gay or lesbian
   3. Bisexual / pansexual
   4. Queer
   5. Not sexual/none
   6. Questioning
   7. Other sexuality
   8. Prefer not to state
   9. Don’t know
6. **What language do you prefer to speak (check all that apply)?**
   1. [ ] English
   2. [ ] Spanish
   3. [ ] Cantonese
   4. [ ] Vietnamese
   5. [ ] Hmong
   6. [ ] Tagalog
   7. [ ] Mandarin
   8. [ ] Korean
   9. [ ] Japanese
   10. [ ] Russian
   11. [ ] Farsi
   12. [ ] Armenian
   13. [ ] Arabic
   14. [ ] Mixteco
   15. [ ] Other: _______________
   16. [ ] Don’t know
   17. [ ] Prefer not to answer

**Are you a health professional?**

______Yes, mental health specialty

______ Yes, other health provider

______ Social/community service provider

______Other provider

______Not a service provider

**Military Service/Veteran Status (check all that apply):**

Current military____ Veteran_____ Family of current military_____ Family member of Veteran______

**Do you have any limitations or disabilities** (Check all that apply):

Communication disabilities:

_____Visual/seeing

_____Hearing

_____Having you speech understood

_____(Other, specify):______________________________________________

Health conditions:

______Serious mental illness

______Other mental health condition

______Physical/mobility

______Chronic Health Condition

______Other (Specify):_____________________________________________

______Declined to Answer

Please check only one box per question:

| **In your opinion…** | Strongly Agree | Agree | Neither Agree or Disagree | Disagree | Strongly  Disagree |
| --- | --- | --- | --- | --- | --- |
| Watching an opera or musical can increase understanding of emotional stress or mental illness and increase empathy |  |  |  |  |  |
| The arts can reduce social stigma of mental illness. |  |  |  |  |  |

Please check only one box per question:

| **Experience of schizophrenia** | YES | NO |
| --- | --- | --- |
| Have you or someone you know ever had schizophrenia or serious mental illness? |  |  |
| Have you supported or provided services for someone with schizophrenia or serious mental illness? |  |  |

Please check only one box per question:

| **How willing would you be to:** | Definitely  Willing | Probably  Willing | Probably  Unwilling | Definitely  Unwilling |
| --- | --- | --- | --- | --- |
| Make friends or socialize with someone suffering from schizophrenia? |  |  |  |  |
| Support or provide services to someone with schizophrenia? |  |  |  |  |

**Positive and Negative Affect Scale (PANAS-X)**

**Please indicate how you feel right now.**

(Use the following scale: 1 (very slightly or not at all), 2 (a little), 3 (moderately), 4 (quite a bit), or 5 (extremely).

Inspired

Proud

Nervous

Distressed

**Positive and Negative Affect Scale (PANAS-X)**

**The Social Connectedness Scale**

(Use the following scale: 1 Strongly Disagree to 6 Strongly Agree.

1. Right now I feel connected to the world around me:
2. Right now I feel that I really belong:
3. Right now I feel less distant from people:
4. Right now I feel a sense of togetherness with my peers:
5. Right now I feel related to other people:
6. Right now I feel connected to society:
7. Right now I feel a sense of brotherhood/sisterhood:
8. Right now I feel I participate with a group:

#### Affect Grid

**Please rate how you feel right now by touching the screen!**


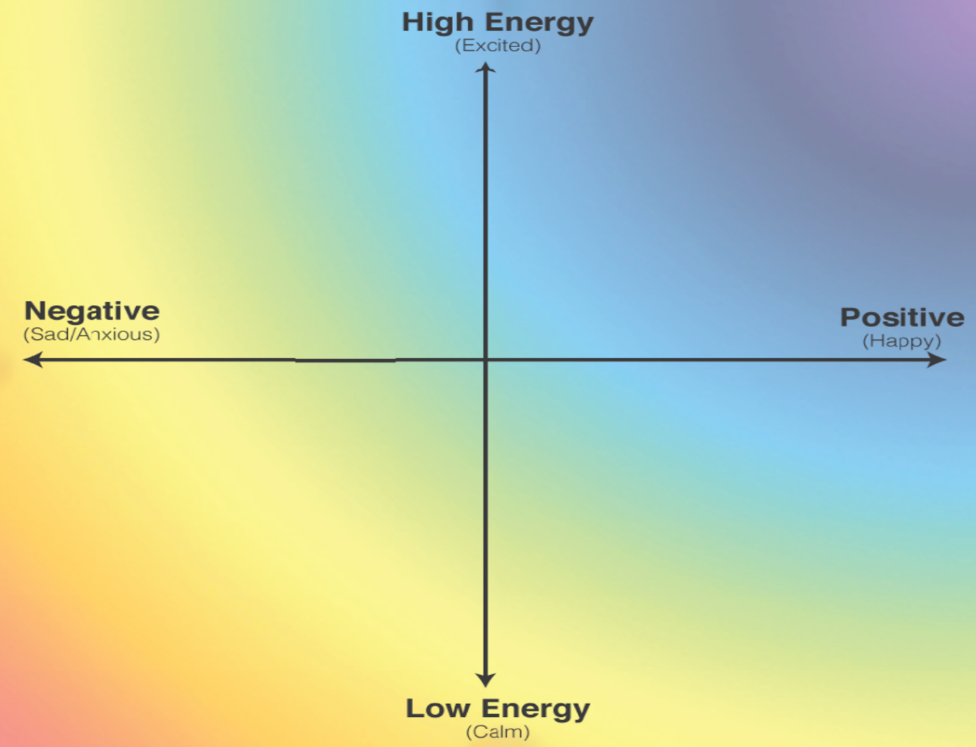


**Thank you! Please save your responses and after the opera complete the post-survey.**

**Please complete the next page after the performance, before leaving (return box or give to staff)**

**Which of the events did you attend? (check all that apply)**

_____Pre-event Workshop on Resilience/Thriving (live, in person)

_____Pre-event Workshop on Resilience/Thriving (remote, through live streaming)

_____Opera Premiere, The Center Cannot Hold Part 2: Recovery (live, in person)

_____Opera Premiere, The Center Cannot Hold Part 2: Recovery (remote, through live streaming)

_____Did not attend any of these events

Please check only one box per question:

| **In your opinion…** | Strongly Agree | Agree | Neither Agree or Disagree | Disagree | Strongly  Disagree |
| --- | --- | --- | --- | --- | --- |
| Watching an opera or musical can increase understanding of emotional stress or mental illness and increase empathy |  |  |  |  |  |
| The arts and social/community events can reduce social stigma of mental illness. |  |  |  |  |  |

Please check only one box per question:

| **How willing would you be to:** | Definitely  Willing | Probably  Willing | Probably  Unwilling | Definitely  Unwilling |
| --- | --- | --- | --- | --- |
| Make friends or socialize with someone suffering from schizophrenia or serious mental illness? |  |  |  |  |
| Support or provide services to someone with schizophrenia or serious mental illness? |  |  |  |  |

Please check only one box per question:

| **Has the event (workshop and opera) moved you in:** | Strongly Agree | Agree | Neither Agree or Disagree | Disagree | Strongly  Disagree |
| --- | --- | --- | --- | --- | --- |
| Being **more** sympathetic towards persons who need mental health services including those with serious mental illness. |  |  |  |  |  |
| Feeling **more** comfortable around persons with serious mental illness. |  |  |  |  |  |
| Feeling **less** alone with concerns about mental illness in yourself or someone you know. |  |  |  |  |  |
| Feeling **more** comfortable talking about mental illness with someone you know. |  |  |  |  |  |
| Reaching out **more** to someone you know to offer support when they are distressed. |  |  |  |  |  |

**On a scale of 1 to 5 with 5 highest, how well did the workshop and opera convey each concept below?**

Workshop on Resilience The Center Cannot Hold Part 2: Recovery

1 2 3 4 5 (high) 1 2 3 4 5 (high)

 Importance of social/family support _______ ______

Importance of hope for recovery _______ ______

A “heroine’s” journey to resilience/recovery _______ ______

Increase understanding/empathy _______ ______

Increase commitment to help others _______ ______

Importance of seeking help or support _______ ______

Personal consequences of social stigma _______ ______

Overall, how satisfied were you with the opera performance?

___Very satisfied

___Somewhat satisfied

___Neutral

___Not very satisfied

___Not at all satisfied

**Positive and Negative Affect Scale (PANAS-X)**

**Please indicate how you feel right now.**

(Use the following scale: 1 (very slightly or not at all), 2 (a little), 3 (moderately), 4 (quite a bit), or 5 (extremely).

Inspired

Proud

Nervous

Distressed

**Positive and Negative Affect Scale (PANAS-X)**

**The Social Connectedness Scale**

(Use the following scale: 1 Strongly Disagree to 6 Strongly Agree.

1. Right now I feel connected to the world around me:
2. Right now I feel that I really belong:
3. Right now I feel less distant from people:
4. Right now I feel a sense of togetherness with my peers:
5. Right now I feel related to other people:
6. Right now I feel connected to society:
7. Right now I feel a sense of brotherhood/sisterhood:
8. Right now I feel I participate with a group:

**Audio Recording (INCLUDE?)**

**We want to hear in your own voice how you feel.  Please push the button to record up to 60 seconds about how you feel right now! Click to record audio 00:00**

#### Affect Grid

**Please rate how you feel right now by touching the screen!**


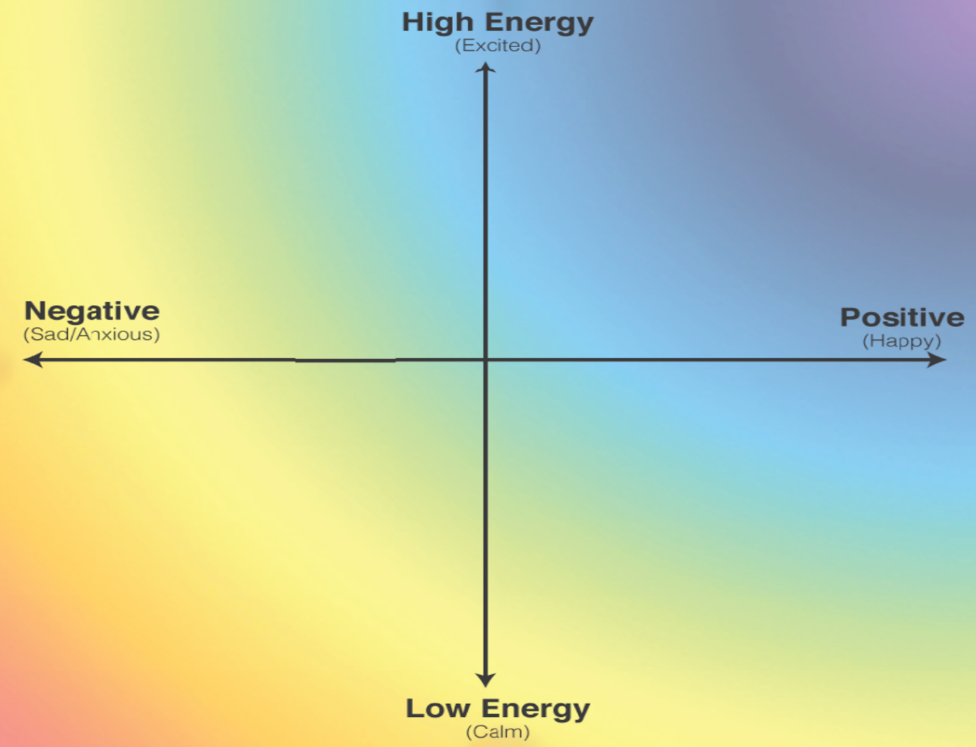


**Thank you! Please save your responses.**

If you would like to share more about how the opera scenes affected you, please share below:
